# Outside testing of wearable robots for gait assistance shows a higher metabolic benefit than testing on treadmills

**DOI:** 10.1038/s41598-021-94448-2

**Published:** 2021-07-21

**Authors:** Florian Leander Haufe, Eléonore Gascou Duroyon, Peter Wolf, Robert Riener, Michele Xiloyannis

**Affiliations:** 1 Sensory-Motor Systems (SMS) Lab, Institute of Robotics and Intelligent Systems (IRIS), ETH Zurich, Zurich, Switzerland; 2 Spinal Cord Injury Center, Balgrist University Hospital, Medical Faculty, University of Zurich, Zurich, Switzerland

**Keywords:** Engineering, Biomedical engineering, Mechanical engineering

## Abstract

Most wearable robots that assist the gait of workers, soldiers, athletes, and hobbyists are developed towards a vision of outdoor, overground walking. However, so far, these devices have predominantly been tested indoors on laboratory treadmills. It is unclear whether treadmill-based laboratory tests are an accurate representation of overground ambulation outdoors with respect to essential outcomes such as the metabolic benefits of robotic assistance. In this study, we investigated the metabolic benefits of the Myosuit, a wearable robot that assists hip and knee extension during the stance phase of gait, for eight unimpaired participants during uphill walking trials in three settings: outside, on a self-paced treadmill with a virtual reality display, and on a standard treadmill at a fixed gait speed. The relative metabolic reduction with Myosuit assistance was most pronounced in the outside setting at − 10.6% and significantly larger than in the two treadmill settings (− 6.9%, *p* = 0.015 and − 6.2%, *p* = 0.008). This indicates that treadmill tests likely result in systematically low estimate for the true metabolic benefits of wearable robots during outside, overground walking. Hence, wearable robots should preferably be tested in an outdoor environment to obtain more representative—and ultimately more favorable—results with respect to the metabolic benefit of robotic gait assistance.

## Introduction

A growing number of wearable robots has been shown to reduce the metabolic cost of human walking and running^1^. Thereby, wearable robots could improve the endurance and load carrying capacity of workers, soldiers, athletes, and hobbyists. Unsurprisingly, these individuals walk and run overground, often outdoors, and not on laboratory treadmills. Yet, most of the promising results presented so far—e.g. 14 out of the 17 studies presented in a recent review^1^—were gathered from participants walking and running on laboratory treadmills at fixed speeds. Here, the implicit assumption is that fixed speed, treadmill-based laboratory testing is a representative rendition of robot-assisted overground ambulation outdoors with respect to metabolic cost. But is this true?

Direct evidence from the field of wearable robots in support of this assumption is scarce. The metabolic cost of walking with some devices has been tested both inside on a treadmill and outside during overground walking^2–4^. However, these studies were not designed to allow for a direct comparison of the results from the inside and outside settings. Kim and colleagues presented their findings in two different publications (outside^2^ and inside^3^). It is unclear if the study population of both projects was identical, if the same assistive settings were used, or in which order outside and inside tests were performed. In another study^4^, the authors did not match the track profile, total elevation change or trial time between inside and outside settings.

In other studies that did not involve wearable robots but more systematically compared the metabolic cost of inside treadmill walking and overground walking^5,6^, treadmill walking was found to incur a higher metabolic cost than overground walking. This increase might be mediated through an altered muscle activation strategy and a resulting difference in the sagittal plane leg joint moments^7^. Since treadmill walking and overground walking are mechanically equivalent at constant speed and regardless of slope^8^, it has been suggested that the root cause of the elevated metabolic cost when walking on a treadmill is the substantially different optic flow that individuals receive on a treadmill compared to overground walking^7^. This explanation is based on earlier observations that humans critically rely on optic flow to achieve efficient motor control strategies during walking^9^. The subjectively perceived balance and surface area of the treadmill might additionally impact the metabolic cost of treadmill ambulation, particularly at higher speeds^10^.

It is unclear to what extent these findings can be transferred to walking with a wearable robot. The additional physical interaction between the human and the robot might confound the motor control of walking on a treadmill. As a result, the metabolic benefit of robotic assistance could change between treadmill and overground settings. Thus, there remains an imminent need to validate whether the metabolic cost of inside treadmill walking with a wearable robot is representative of overground walking with the same robot in an outdoor environment.

**Figure 1:**
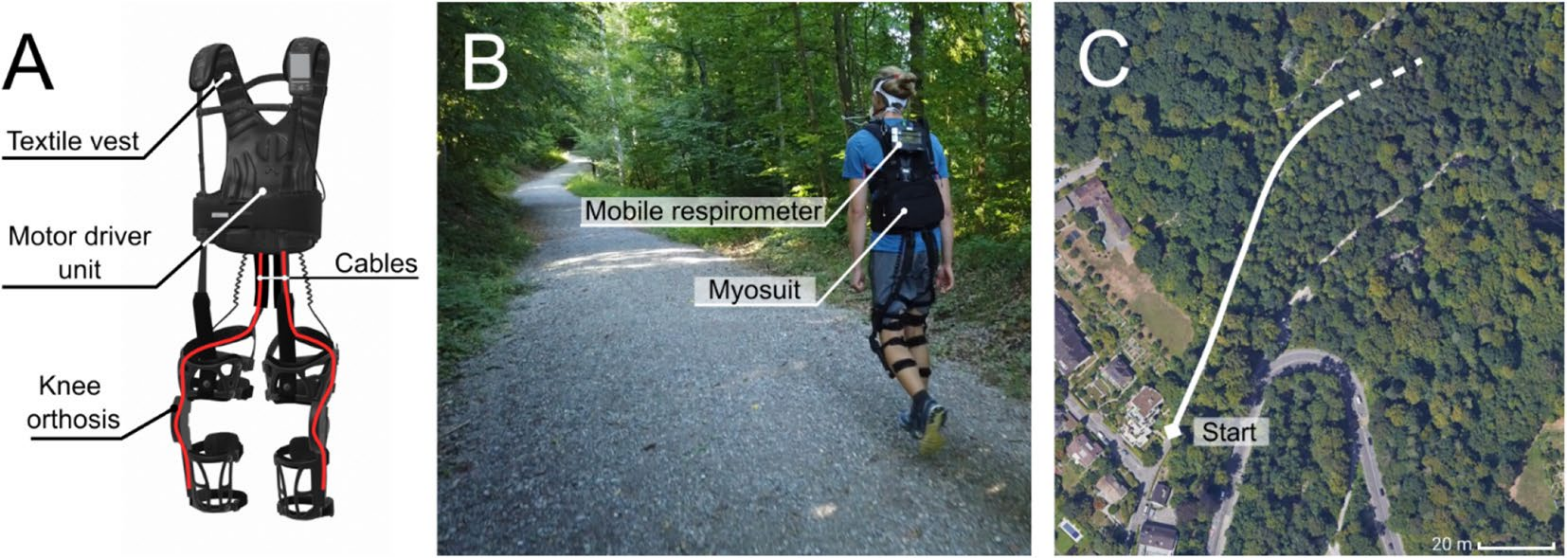
(**A**) Architecture of the Myosuit with the drive cables marked in red. (**B**) Example picture of a participant wearing the Myosuit and the mobile respirometer in the OUT setting. (**C**) Aerial image of the OUT setting with the path and start marked in white. The total length of the path depended on the participants’ individual walking speeds.

In the present study we have addressed this topic. As an example wearable robot, we used the Myosuit (see Fig. 1). The Myosuit assists walking in basic mechanical functions^11^ by supporting the user’s bodyweight and progression from weight acceptance into late stance. On each leg, one cable is routed across the hip and knee joints—exploiting natural extension synergies^12^—and works in parallel with the muscles which have the largest contribution to bodyweight support during walking^13^. In previous work from our group, assistance from the Myosuit has been shown to reduce the metabolic cost of uphill walking compared to wearing the suit in zero-force mode (i.e. with assistance disabled) on a fixed-speed treadmil^14^.

**Figure 2:**
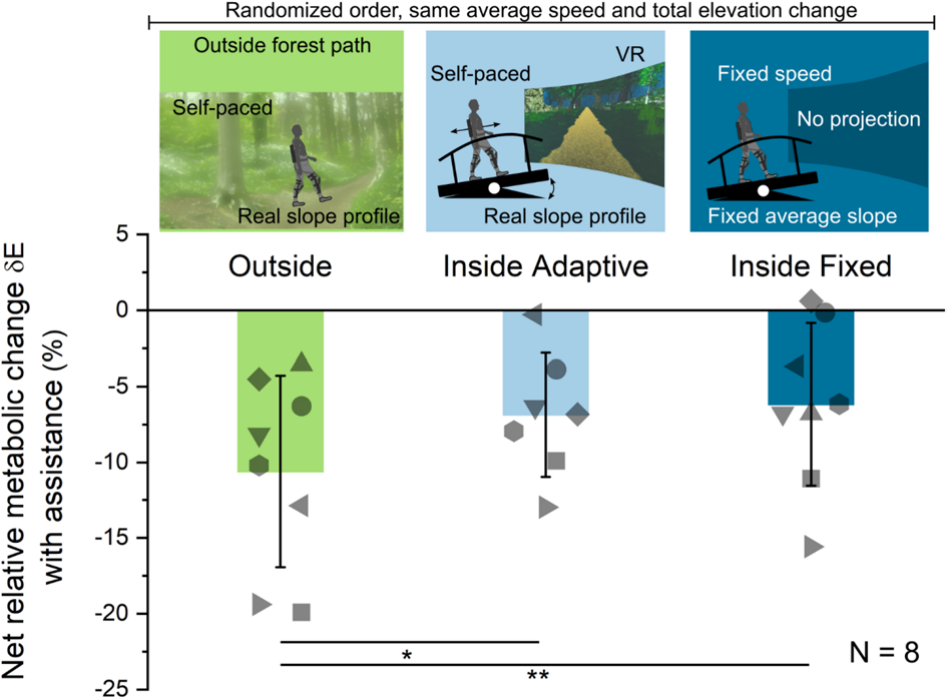
Net relative metabolic change when walking with Myosuit assistance compared to walking with the Myosuit in zero-force mode. Three settings were investigated: outside, inside adaptive and inside fixed with eight participants. For the inside adaptive condition, data from one participant was corrupted. Significant differences between the outside and both inside conditions were identified through analysis with a linear mixed effects model. Error bars are ± 1 standard deviation.

In the present study, we analyzed the metabolic cost of walking with Myosuit assistance in three different settings for eight participants. As reference setting, we considered overground walking on an outside forest path (“OUT”, see Fig. 2). Secondly, we included a treadmill setting that represents the state-of-the-art of movement laboratory testing (“IN_Adapt_”, see Fig. 2). In this setting, we used marker-based self-pacing, rendered the altitude profile of the outside reference path by dynamically pitching the treadmill, and presented a 180° virtual reality (VR) display of an outside forest path that was synchronized to the participant-driven treadmill speed (see Methods). A third setting, referred to as “IN_Fix_”, represents the setup commonly found in more basic lab tests of wearable robots. The treadmill ran at a fixed belt speed at a fixed average pitch and no visual display was provided (see Fig. 2).

As primary study outcome, we have analyzed the net relative metabolic change with wearable robotic assistance, δE, compared to walking with the robot in zero-force mode. The relative metabolic change with assistance, either compared to wearing the robot in a variation of zero-force mode or to not wearing the robot at all, is by far the most frequently used metric to present the metabolic effects of wearable robots^1,15^.

To define δE for our analysis, we introduce the following gross metabolic consumptions: during quiet standing as E_rest_, during walking in zero-force mode as E_zero_, and during walking with assistance as E_assist_. Based thereon, we define δE as

1 $$\updelta E=\frac{{E}_{assist}-{E}_{zero}}{{E}_{zero}-{E}_{rest}}=\frac{\Delta E}{{E}_{zero,net}}$$

Here, ΔE is the absolute metabolic change with assistance and E_zero,net_ is the net metabolic rate in the zero-force condition, where “net” indicates that E_rest_ has been subtracted from the gross (i.e. as-measured) consumptions.

We hypothesized that during outside, overground walking, δE would be larger—corresponding to a more pronounced metabolic benefit from Myosuit assistance—than when walking in the two inside treadmill settings. To assess high-level changes in the participants’ gait patterns as confounding factors, we analyzed stride times and stride time variabilities. The participants’ gender was modelled in our analysis, to account for potential differences between females and males, by including a categorical fixed effect predictor variable “gender” in a linear mixed effects model (details in Statistical Analysis).

## Results

### Metabolic cost

The net relative metabolic change with assistance compared to zero-force mode δE was most pronounced during outside overground walking (OUT δE = −10.6%) and significantly larger than in both of the treadmill-based settings (IN_Adapt_ δE = −6.9%, t(18) = 2.7, *p* = 0.015, IN_Fix_ δE = −6.2%, t(18) = 3.0, *p* = 0.008, see Fig. 2 and Table 1). For the five male participants, δE was less pronounced than for the three female participants (t(18) = 3.0, *p* = 0.007, see Table 1).

**Table 1:** Statistical model fit results for the primary study outcome δE, the net relative metabolic change with assistance in %.

Coefficient name	Estimate (%)	95% CI lower	95% CI upper	t	*p*
(Intercept)	− 16.4	− 24.7	− 8.2	− 4.2	< 0.001
Gender: Male	6.7	2.1	11.4	3.0	0.007
Speed	1.5	− 6.5	9.4	0.4	0.70
Setting: IN_Adapt_	4.1	0.9	7.4	2.7	0.015
Setting: IN_Fix_	4.4	1.3	7.5	3.0	0.008
Random Effect Covariance	2.0	0.9	4.9		
Residual Standard Error	3.0	2.1	4.2		

**Figure 3:**
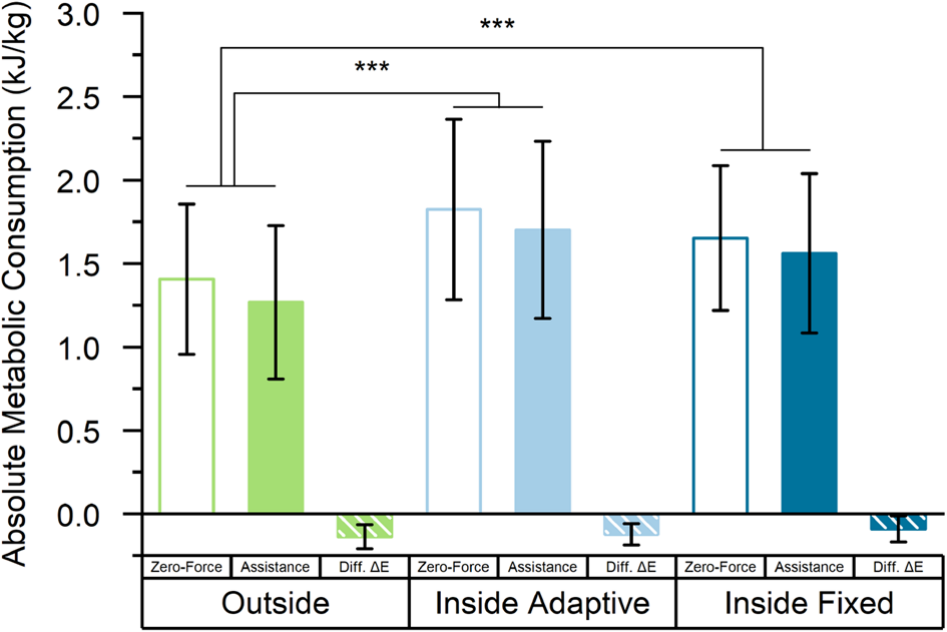
Absolute metabolic consumption when walking with the Myosuit in zero-force mode and when walking with Myosuit assistance in the OUT, IN_Adapt_ and IN_Fix_ setting over a 4-min trial. The absolute difference between assistance and zero-force metabolic consumption, ΔE, is shown as well. Data is averaged over all participants and error bars are ± 1 standard deviation. Significant differences between the OUT and the two IN settings were identified.

In parts, the observed differences in δE resulted from differences between settings in the metabolic consumptions E_zero,net_ and E_assist,net_. In the two IN settings, metabolic consumptions were generally higher than in the outside setting (effect size for IN_Adapt_ was 430 J/kg, t(40) = 13.9, *p* < 0.001, for IN_Fix_ 270 J/kg, t(40) = 9.2, *p* < 0.001, see Fig. 3 and Table 2). There was no difference between genders with respect to the metabolic consumption (t(40) =  −0.3, *p* = 0.75, see Table 2).

**Table 2:** Statistical model fit results for the absolute metabolic consumption E during 4-min walking trials in J/kg.

Coefficient name	Estimate (J/kg)	95% CI lower	95% CI upper	t	*p*
(Intercept)	− 231	− 595	133	− 1.2	0.21
Gender: Male	− 34	− 242	175	− 0.3	0.75
Speed	1490	1134	1846	8.5	< 0.001
Setting: IN_Adapt_	430	368	493	13.9	< 0.001
Setting: IN_Fix_	270	210	330	9.2	< 0.001
Condition: Assistance	− 117	− 167	− 67	− 4.8	< 0.001
Random Effect Covariance	119	70	203		
Residual Standard Error	83	66	104		

Another portion of the observed differences in δE was caused by changes in ΔE, the absolute metabolic change with Myosuit assistance, between settings, although these were not significantly different (OUT ΔE = −138 J/kg compared to IN_Adapt_ ΔE = −122 J/kg, t(18) = 0.9, *p* = 0.40, IN_Fix_ ΔE = −92 J/kg, t(18) = 2.1, *p* = 0.054). Yet, a trend towards ΔE being smaller in the IN_Fix_ setting might be hinted (see also Fig. 3). For male participants, ΔE was smaller than for females (t(18) = 3.3, *p* = 0.004).

### Gait characteristics

**Figure 4:**
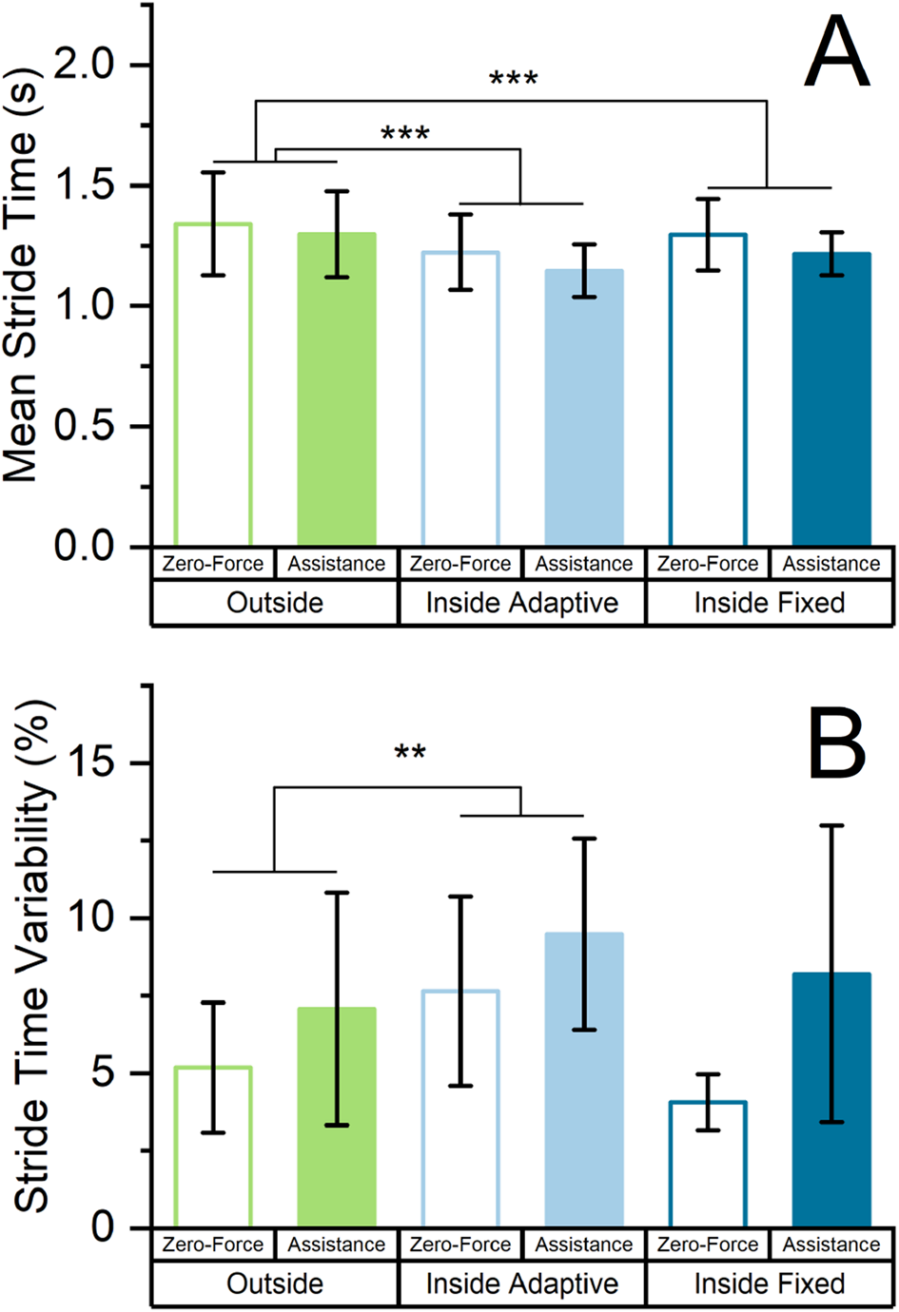
(**A**) Mean stride times for the three settings when walking in zero-force mode and with assistance, averaged over all participants. Stride times were significantly shorter in both inside treadmill settings. (**B**) Stride time variability presented as the coefficient of variation, averaged over all participants. The stride time variability was higher in the inside adaptive setting than in the outside setting. All error bars are ± 1 standard deviation.

The mean stride times of the IN_Adapt_ and IN_Fix_ settings were shorter than in the OUT setting (t(38) =  −6.6, *p* < 0.001 and t(38) =  −4.7, *p* < 0.001, respectively, see Fig. 4A). Further, mean stride times were shorter with assistance compared to when walking in zero-force mode (t(38) =  −4.2, *p* < 0.001) and with increasing walking speed (t(38) =  −3.2, *p* = 0.003).

Stride time variability—represented as coefficient of variation—was higher in the IN_Adapt_ treadmill setting compared to outside walking (t(38) = 3.3, *p* = 0.002, see Fig. 4B). There was no difference between the IN_Fix_ setting and outside walking (t(38) =  −0.2, *p* = 0.81). Stride time variability was higher with assistance compared to walking in zero-force mode (t(38) = 4.3, *p* < 0.001) and did not vary with speed (t(38) =  −1.3, *p* = 0.21).

Complete statistical results for the gait characteristics are included in the Supplementary Material (Table S2 and S3).

## Discussion

### Relative metabolic benefit of assistance is higher outside

If this had been a typical treadmill-based laboratory study, we would have concluded that Myosuit assistance reduced the metabolic cost of uphill walking by around 6 to 7% compared to wearing the Myosuit in zero-force mode. However, with this study’s additional outdoor setting, we observed a significantly larger metabolic cost reduction of about 10% during outside, overground walking with the Myosuit.

As a result, there is a 4.1% difference between the relative metabolic reduction found in the IN_Adapt_ setting and the one observed in the OUT setting (4.4% for IN_Fix_). The respirometer used in this study had an intra-device minimum detectable difference (MDC) of less than 2.6%^16^. Beyond the MDC, the practical relevance of changes in the metabolic cost of walking is closely linked to the specific application but at times differences as low as 3.3% are considered practically relevant^17^.

Thus, the effect of the test setting on the relative metabolic reduction δE with Myosuit assistance exceeds both the MDC and the threshold above which differences are considered practically relevant. The contributions underlying this effect are discussed in the subsequent sections.

### Contribution of the absolute metabolic consumption

In line with previous work^5,6^, we found that the metabolic consumptions E_zero,net_ and E_assist,net_ were higher in the IN_Adapt_ and IN_Fix_ settings than in the OUT setting (see Fig. 2). This increase in the metabolic consumption during treadmill walking contributed to the decreased magnitude of δE in the IN settings. It has been attributed to differences in the optic flow that in turn elicit a change in the muscle activation strategy and the resulting joint moments^7^. An increase in the metabolic consumption was expected for IN_Fix_ without any visual projection based on previous literature^7^ but also observed for IN_Adapt_. Hence, the VR projection shown in the IN_Adapt_ setting might have failed to provide optic flow that was representative of outside walking. The VR projection was limited with respect to the vertical coverage since the screen height was substantially smaller than vertical field of view, particularly if participants angled their view downwards towards the treadmill. In addition, we cannot rule out a minor delay between the treadmill de- and accelerations and the VR projection that might have degraded the coherence of the optical and remainder sensory input of participants. For future experiments, it might be worthwhile to test the use of augmented reality head-mounted displays that can achieve a better coverage for optic flow, although these devices have been shown to affect the dynamics of treadmill walking themselves^18^ and will require familiarization.

The IN_Adapt_ setting was also associated with a decreased mean stride time (see Fig. 4A) and an increased stride time variability (see Fig. 4B) compared to the OUT setting, and showed the highest absolute metabolic consumption of all three settings (see Fig. 3). We attribute this to the interaction between the participants and the self-pacing algorithm of the treadmill. To a varying degree, participants were forced to walk towards the front of the treadmill to avoid unintentional deceleration. In this position, the front end of the belt was likely perceived as uncomfortably close, effectively resulting in shorter steps that were more variably positioned in response to the behavior of the self-pacing algorithm. This might have resulted in a scenario where participants felt only partially able to walk freely, and in parts, felt constrained by the self-pacing algorithm, its latency, and implicit concerns about walking too close to the end of the treadmill.

Both shorter than self-selected stride length^19^ and increased step length variability^20^ have been independently associated with an increased metabolic cost of walking, as was an externally enforced stepping pattern^21^. Hence, the IN_Adapt_ setting might have—despite being designed with the goal of adaptively accommodating for user-driven gait patterns—confounded temporal gait characteristics most strongly out of the three tested settings. An interference of the IN_Adapt_ setting with normal walking might also be evidenced in the participants’ rating of perceived exertion. In the IN_Adapt_ setting, participants tended to rate their exertion higher than proportionally expected based on a linear regression model with respect to their metabolic consumption (see Supplementary Material Figure S1). The disproportionately elevated perceived exertion could hint towards a reduced feeling of safety or increased cognitive load during walking.

All study participants were presumably familiarized with treadmill walking after completing a familiarization session comprising a total of 32 min of walking, considerably more than the 6 min previously reported as required^22^. However, it is important to note that our familiarization session did not include walking in the IN_Adapt_ setting with the self-pacing activated due to the considerable setup overhead. It seems as if such a dedicated familiarization will be required to (potentially) profit from the purported benefits of self-paced treadmill walking. We cannot rule out that with specific familiarization to the IN_Adapt_ setting, results would differ.

### Contribution of the change in consumption with robotic assistance

While not significant themselves, the observed differences in the absolute metabolic change ΔE between the IN_Adapt_ and IN_Fix_ settings and the OUT setting did contribute to the significantly decreased magnitude of δE in the two IN settings.

An analysis of the 95% confidence intervals (CI) of the model coefficients encoding the IN_Adapt_ and IN_Fix_ settings (see Table 3) indicates that in these two settings, the absolute metabolic change with assistance is not more pronounced than in the outside setting. Here, we consider that a relative MDC of 2.6% would correspond to an absolute MDC of 41 J/kg for the average metabolic consumption measured in this study. The coefficients’ lower 95% CI bounds of −29 J/kg (IN_Adapt_) and −0.8 J/kg (IN_Fix_, see Table 3) are contained within an interval of [- MDC, MDC] or [− 41, 41] which we would consider equivalent to the OUT setting. On the other hand, the upper bounds of the 95% CI of 70 J/kg and 94 J/kg fall outside of this interval and hence leave it uncertain if ΔE might be less pronounced in the IN settings than in the OUT setting. Hence, we can follow that testing on treadmills will not meaningfully overestimate the absolute metabolic benefit of robotic assistance during outside, overground walking, but might perhaps understate it.

**Table 3:** Statistical model fit results for the absolute metabolic change ΔE with assistance in J/kg.

Coefficient name	Estimate (J/kg)	95% CI Lower	95% CI Upper	t	*p*
(Intercept)	− 154	− 265	− 44	− 2.9	0.009
Gender: Male	99	36	161	3.3	0.004
Speed	− 41	− 147	64	− 0.8	0.42
Setting: IN_Adapt_	20	− 29	70	0.9	0.40
Setting: IN_Fix_	47	− 0.8	94	2.1	0.054
Random Effect Covariance	24	8	76		
Residual Standard Error	45	32	64		

### Implications for the field

In summary, we observed an increase in the absolute metabolic consumption during treadmill walking and found that absolute metabolic reduction due to robotic assistance on a treadmill is at most as large as during outside walking. Thus, the relative metabolic change δE during treadmill walking will be systematically lower than the true relative metabolic change that would be found during outside, overground walking.

This observation represents a strong incentive for the developers of wearable robots to test their devices in outdoor environments during overground walking. Outdoor testing will likely show a larger metabolic benefit of wearable robotic assistance and hence be highly desirable for developers and researchers in the field. At the same time, the current practice of testing wearable robots on treadmills at fixed walking speeds appears justified given that the metabolic effects observed during these inside tests will be a systematically low estimate for the true effects in the target outdoor, overground setting.

In this study, the added technical complexity of the IN_Adapt_ setting did not result in a more lifelike rendition of outdoor overground walking with respect to temporal gait characteristics, and probably neither with respect to optic flow. On the contrary, we observed a pronounced interference between the self-pacing mechanism and the participants’ ability to follow their own stepping pattern. It remains to be seen if with prior user familiarization and further tuning of this setting can improve upon standard, fixed treadmill testing. Without further evidence, it seems preferrable to rely on such a simpler treadmill setup or directly transition to where the most favorable and representative results will be found—outside.

## Methods

### Participants

For this study, eight unimpaired participants (3 female, 5 male) with a mean age of 25.8 yrs (range 23—29 yrs), mean body mass of 74 kg (60—97 kg) and mean height of 180 cm (163–199 cm) were recruited through referrals. Being unimpaired was defined as the absence of any musculoskeletal and neurological impairments related to gait function and cardiovascular limitations that would prevent prolonged walking at elevated speeds. Written informed consent to participate in this study and to the publication of their images in an online open-access publication was obtained from the participants prior to the experiments. The study design and protocol were approved by the institutional review board of ETH Zurich (EK 2019-N-172) and performed in accordance with the Declaration on Helsinki.

### Wearable robot

The wearable prototype used in this study (Myosuit Beta, MyoSwiss AG, Switzerland) was designed to assist weight-bearing and forward/upward progression during the stance phase of walking (see also previous work^14^).

The Myosuit comprised a backpack-style motor driver unit that housed two electric motors with reduction gears, a battery, and the control electronics (see Fig. 1). On each leg, a pulley cable was routed from the driver unit posteriorly across the hip joint, laterally across the thighs, and frontally crossed the knee joint supported by a cam. The cables were anchored to a 3D-printed polymer knee orthosis attached to the thigh and shank. Cables were made from ultra-high molecular weight polyethylene.

Inertial Measurement Units (IMUs) were placed on both shank and thigh segments and in the motor driver unit to measure linear accelerations and rates of rotation. Based on the IMU sensor data, limb angles and trunk posture were estimated using a five-segment body model. Heelstrike and toe-off events were detected using a previously described algorithm^23^ and used in conjunction with joint angle estimates to time the cyclic onset and duration of assistive forces.

A textile upper body vest with a waist belt was used to interface the motor driver unit and the knee orthoses to the participants. Two passive elastomer springs that frontally crossed the hip joint were only marginally tensioned to counteract downward slipping of the knee orthoses.

During the experiments, two different control modes were used: Assistive mode and transparency.

In assistive mode, the Myosuit actively supports weight-bearing during the stance phase of walking^24^. Shortly after heel strike (detected by the IMU on each shank) of each leg, the ipsilateral tendon of the robotic device is tensioned at a rate that is modulated by cadence and reaches a magnitude that is proportional to the momentary knee angle, where higher cadences result in higher rates of force application and more knee flexion results in a higher magnitude. Such tension on the cable is held until the hip angle crosses a threshold that, by default, is set to be 0° (femur parallel to gravity) but can be modulated by the user, to shorten or extend the duration of assistance. Upon crossing said threshold, the robotic tendons are slacked, releasing the leg and seamlessly allowing it to advance forward to the swing phase of the gait cycle, until the subsequent heel-strike. The parameters that modulated the magnitude and duration of assistance where chosen based on previous human-in-the-loop optimization experiments with a pilot participant representative of this study’s population^25^, resulting in peak forces of 212 N and assistance between 10 and 42% of the gait cycle. In transparency mode, the device is controlled to keep a low, close-to-zero interaction force (hence the “zero-force” condition of our study) with its user, with the goal of not hindering human movement. This is done using IMU measurements and motor encoder data to drive an array of compensation components. These components partially compensate for the (undesirable) interaction forces that arise from changes in the limb position and velocity, from the compliance of human tissue, from friction, from inertia and from the series elasticity of the transmission. While interaction forces with the human wearer are ideally close to zero when in transparency mode, the linear cable forces are generally non-zero (< 20 N), to avoid slack and discontinuities^26^.

## Study design

### General

The participants each completed one familiarization session and subsequently two study sessions, one inside in the laboratory where two experimental settings (IN_Fix_ and IN_Adapt_) were tested and one outside with one experimental setting (OUT). The order of execution was pseudo-randomized such that, to the extent mathematically possible, the same number of participants started with each of the three settings. Due to practical considerations, the IN_Fix_ and IN_Adapt_ setting were always tested consecutively in one session, i.e. OUT was never tested in second position but always first or last. Between the adjacent IN blocks, participants took a ten min break to rest.

Within each experimental setting, participants started with a four-minute block of quiet standing during which their metabolic consumption at rest was approximated. Afterwards, the participants completed four consecutive walking trials, each lasting four minutes with breaks of two minutes in between. To reduce the effect of fatigue, trials were performed in the order “zero-force”, “assistance”, “assistance”, “zero-force” and averaged over the two repetitions. After each trial, the participants were asked to rate their perceived exertion using an adapted Borg Scale^27^.

### Familiarization

In the familiarization session, participants walked on the treadmill (V-Gait Dual Belt, Motekforce Link, The Netherlands) while wearing the Myosuit in zero-force and assistance mode at various fixed speeds and pitches for eight consecutive four-minute trials. At the end of the session, the speed the individual participant felt confident to be able to maintain over 40 min of walking was determined by iterative feedback and adjustment. No measurements were taken in this session.

### Outside (OUT)

Outside tests were conducted on a gravel uphill trail located at 47°21′47.6"N 8°34′26.0"E on the outskirts of Zurich, Switzerland. The path was well-sheltered from wind and nominal wind speeds during tests were always below 4.2 m/s. More detailed information in the weather conditions during individual visits is presented in the Supplementary Material (Table S1). Orange security cones were placed at 25 m intervals along the trail (see Supplementary Video V1). The participants were provided audio cues with a portable speaker at fixed time intervals and asked to choose their walking speed such that on average, the cues coincided with them passing the cones. The timing of the audio cues was calculated to pace the participants at their self-selected walking speed from the familiarization session. In between trials, participants were given sufficient time to walk down the gravel trail to the start and rest for at least two minutes.

### Inside adaptive (IN_Adapt_)

In the inside adaptive setting, a set of 16 passive reflective markers was placed on the participants’ legs and the Myosuit (marker placement adapted from “Plug-In Gait Lower-Limb”-model, Nexus, Vicon, UK) and tracked with an array of 10 infrared cameras (Bonita B10, Vicon, UK). The markers were used to determine the longitudinal position of the participant on the treadmill and to adjust the belt speed accordingly in a real-time feedback loop. The feedback loop, adopted from the work of Sloot and colleagues^28^, consisted of a corrected close-loop PD-controller of the form:

$$u = P\Delta x- \Delta xD\Delta \dot{x}$$

with ∆x being the difference between the position of the participant and the midline of the treadmill in the longitudinal direction, ∆x ˙ its time derivative and u the control signal, representing a speed correction in the form of an ac-/deceleration for the motors of the treadmill. The position of the participant in the longitudinal direction was estimated from the average position of four pelvic markers, low-pass filtered at 2 Hz. When the participant accelerated and hence initially moved towards the front of the treadmill, the belt speed was increased, and vice versa when the participant decelerated. This enabled the participants to voluntarily adjust their walking speed. In addition, the pitch of the treadmill was continuously adapted relative to the distance the participant had covered and matched to the slope profile of the outside path. During pilot tests, this slope profile was approximated from several GPS tracks (see Supplementary Material Figure S2). In front of the treadmill, a virtual environment resembling an outside path was projected on a 180° curved screen (Gait Real-time Analysis Interactive Lab (GRAIL) system, Motekforce Link, The Netherlands). Within this projection, a digital number was shown for a duration of 5 s every 25 m (see also Supplementary Material Video V2). The number indicated the cumulative distance participants lagged (negative sign) or lead relative to their self-selected speed thus far through the trial.

### Inside fixed (IN_Fix_)

In this setting, the treadmill speed was fixed to the participants’ self-selected speed and the pitch was fixed to the corresponding average pitch of the outdoor trail (see Supplementary Video V3). The average pitch depended on the self-selected speed as the slope profile of the outdoor trail was not uniform.

### Measurement setup

#### Metabolic measurements

Breath-by-breath respiratory data was collected using a portable respirometer (K5, COSMED, Italy) to estimate the metabolic cost using Péronnet's formula^29^. The participants were asked to fast and drink only water at least eight hours prior to experimental sessions to limit the influence of the digestive metabolism and obtain a reliable estimate of their metabolic costs. The respirometer was calibrated prior to each experimental session by performing, in sequence: a flowmeter calibration using a 3 $l$ calibration syringe; an O2 and CO2 sensor calibration using a reference gas with known concentrations of the two gasses (CO2 5%, O2 16%); a calibration of the delay between flow and gas measurements, performed through time-matched inspiration/expiration cycles.

#### Stride time estimation

The stride times were calculated based on a Kalman filter estimate of the shank angles measured by the integrated IMUs of the Myosuit using the algorithm described in Grimmer et al*.*^23^. There, we showed that step segmentation using this approach can lead to a noticeable bias in time but excellent precision, with the addition of empirical rule sets. We defined the shank angle as the angle between the horizontal plane and the primary axis of the shank segment. A stride was then defined as the period between consecutive local maxima of the shank angle estimate of one leg. The coefficient of variation, being independent of the measurement unit, was used to compare data sets with different means across participants.

### Statistical analysis

A linear mixed effects model was fitted to our primary study outcome δE and the secondary outcomes E, ΔE, the mean stride time and the coefficient of variation of the stride time using least squares regression (Matlab, USA).

For the two differential outcomes δE and ΔE, the model included two reference-dummy-encoded, categorical fixed effect predictor variables, “setting” (possible values: OUT (reference), IN_Adapt_, IN_Fix_) and “gender” (female (reference), male), a continuous fixed effect variable “speed” in units of m/s, a random effect variable “participant” and the intercept. The choice of reference levels for the dummy-encoded setting variable was made a priori to obtain the desired contrasts OUT- IN_Adapt_ and OUT- IN_Fix_. For the non-differential outcomes, an additional categorical predictor variable “condition” with values (zero force (reference), assistance) was included in the model. Following prior model analysis, no interaction terms were included.

## Supplementary Information

Supplementary Information 1.

Supplementary Video 1.

Supplementary Video 2.

Supplementary Video 3.

## Data Availability

All data generated and analysed during this study are included in this published article and its Supplementary Material files.
