# Supplementary material for: Outside testing of wearable robots for gait assistance shows a higher metabolic benefit than testing on treadmills
Source: Sci Rep. 2021 Jul 21;11:14833. doi: 10.1038/s41598-021-94448-2 (PMC8295285; doi:10.1038/s41598-021-94448-2)
Supplement: Supplementary file 1 — Supplementary Information 1. [file 41598_2021_94448_MOESM1_ESM.docx]

Supplementary Material

# Rating of perceived exertion

Figure S1. Perceived effort determined through the Borg Scale Rating as a function of the net metabolic consumption over the 4-minute walking trials determined through indirect calorimetry. Symbol colors encode the setting, symbol fill encodes zero-force and assistance, and symbol shape the individual participants. A least-squares linear regression (dashed line) indicates a significant, moderate correlation between perceived effort and net metabolic consumption

The perceived effort determined through the Borg Scale Rating was correlated with the net metabolic consumption (see Figure 4). Visual analysis of the data suggests that based on a linear regression estimate with respect to their net metabolic consumption, participants tended to perceive their effort as lower than expected when walking with assistance and when walking outside. Conversely, they perceived their effort as higher than proportionally expected when walking in zero-force mode and in the IN_Adapt_ setting.

# Weather conditions during outside tests

Table S1. Weather conditions during the outside tests for the individual participants. There was no precipitation during any of the outside tests.

| **Participant**  **ID** | **Conditions** | **Ambient Pressure (hPa)** | **Temperature (°C)** | **Relative humidity (%)** |
| --- | --- | --- | --- | --- |
| P1 | fair, cloud cover | 965 | 16 | 50 |
| P2 | sunny | 948 | 18 | 69 |
| P3 | sunny | 960 | 21 | 64 |
| P4 | sunny | 963 | 24 | 61 |
| P5 | fair, cloud cover | 955 | 22 | 71 |
| P6 | sunny, partially cloudy | 957 | 20 | 63 |
| P7 | sunny | 957 | 25 | 72 |
| P8 | sunny | 959 | 23 | 60 |

For reference, the temperature during inside tests was between 21 and 23 °C and relative humidity was between 60 and 70%. These conditions were maintained by forced ventilation and when necessary, automated air conditioning.

# Missing data and experimental errors

For participant P3, an incorrect slope profile was used during tests in the IN_Adapt_ setting and the respective measurements were excluded from our analysis. This explains the missing data point in Figure 2 of the main manuscript, where only 7 entries are shown for the “Inside Adaptive” setting. Further, for participant P7, the forest path VR projection comprised in the IN_Adapt_ setting was erroneously also displayed in the IN_Fix_ condition. Considering the minor effect of the projection evidenced in the remainder participants, we decided to still include the respective measurements in the overall analysis.

The IMU data underlying our calculation of stride time and stride time variability was corrupted or lost because of experimenter error for participant P1 in trial 1 (zero-force) of the IN_Adapt_ setting and for P5 in trial 1 (zero-force) of the IN_Fix_ setting. For these participants, the final outcome metrics for the zero-force condition – usually calculated as averages of the zero-force trials 1 and 4 – were only based on the results from trial 4. For participant P2, the entire IMU data set for the IN_Fix_ setting was lost and hence was not available for the overall analysis.

The Borg Scale Ratings of participant P8 in the OUT setting were not recorded.

# Figures


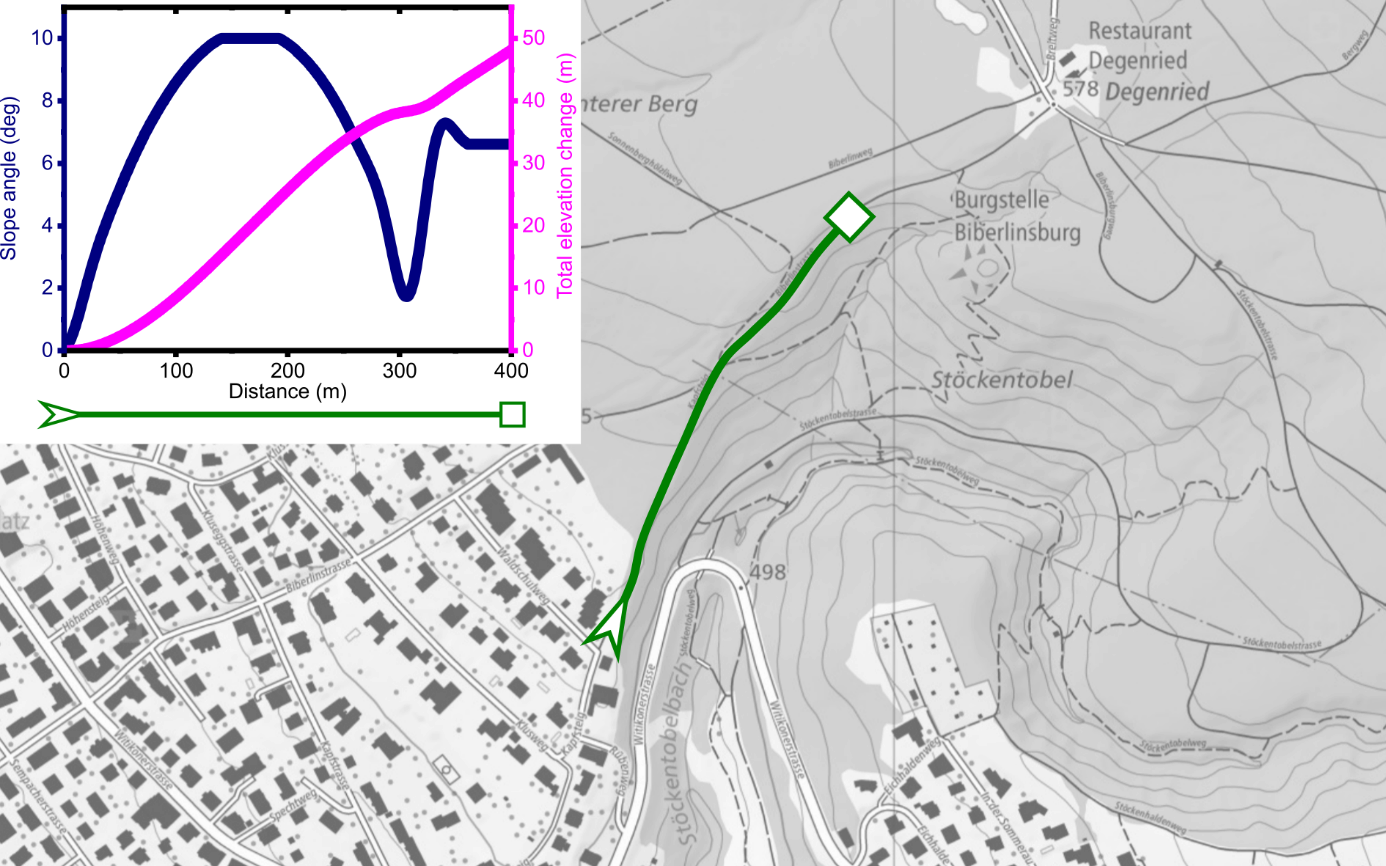

Figure S2. Map view of the outside path participants walked on in the OUT setting. The inset shows the slope angle (as replicated on the treadmill in the IN_Adapt_ setting) and the cumulative total elevation change relative to the start of the path. Slope data has been calculated based on filtered GPS data from several pilot runs and compared against federal map data for validation.

Figure S3. Normalized distance covered in the three settings. The mean for the OUT condition is set to 100% and the standard deviation represents a ±5 m margin within which all participants finished all trials in the OUT setting. Data for the two IN settings is based on millimeter-exact recordings of the treadmill belt distance for each participant and trial. Throughout the three settings, the distance covered by participants was very similar.

# Supplementary Videos

**V1** Participant walking in OUT setting

**V2** Participant walking in IN_Adapt_ setting

**V3** Participant walking in IN_Fix_ setting

# Tables

Table S2. Statistical model fit results for the mean stride time in units of ms.

| **Coefficient Name** | **Estimate (ms)** | **95% CI Lower** | **95% CI Upper** | **t** | **p** |
| --- | --- | --- | --- | --- | --- |
| (Intercept) | 1782 | 1528 | 2035 | 14 | < 0.001 |
| Gender: Male | - 5 | - 149 | 139 | - 0.1 | 0.95 |
| Speed | - 385 | - 632 | - 138 | - 3.2 | 0.003 |
| Setting: IN_Adapt_ | -127 | - 167 | - 88 | - 6.6 | < 0.001 |
| Setting: IN_Fix_ | - 91 | - 130 | - 51 | - 4.7 | < 0.001 |
| Condition: Assistance | - 66 | - 98 | - 34 | - 4.2 | < 0.001 |
| Random Effect Covariance | 83 | 49 | 140 |  |  |
| Residual Standard Error | 52 | 42 | 66 |  |  |

Table S3. Statistical model fit results for the coefficient of variation of the stride time in %.

| **Coefficient Name** | **Estimate (%)** | **95% CI Lower** | **95% CI Upper** | **t** | **p** |
| --- | --- | --- | --- | --- | --- |
| (Intercept) | 9.5 | 4.2 | 14.8 | 3.6 | < 0.001 |
| Gender: Male | - 1.9 | - 4.8 | 1.0 | - 1.3 | 0.20 |
| Speed | - 3.2 | - 8.3 | 1.9 | - 1.3 | 0.21 |
| Setting: IN_Adapt_ | 2.5 | 1.0 | 4.0 | 3.3 | 0.002 |
| Setting: IN_Fix_ | 0.2 | -1.7 | 1.3 | - 0.2 | 0.81 |
| Condition: Assistance | 2.6 | 1.4 | 3.8 | 4.3 | < 0.001 |
| Random Effect Covariance | 1.5 | 0.8 | 2.9 |  |  |
| Residual Standard Error | 2.0 | 1.6 | 2.5 |  |  |
